# Estrogen-Mediated Renoprotection following Cardiac Arrest and Cardiopulmonary Resuscitation Is Robust to GPR30 Gene Deletion

**DOI:** 10.1371/journal.pone.0099910

**Published:** 2014-06-12

**Authors:** Michael P. Hutchens, Yasuharu Kosaka, Wenri Zhang, Tetsuhiro Fujiyoshi, Stephanie Murphy, Nabil Alkayed, Sharon Anderson

**Affiliations:** 1 Department of Anesthesiology & Perioperative Medicine, Oregon Health & Science University, Portland, Oregon, United States of America; 2 Division of Nephrology and Hypertension, Oregon Health & Science University, Portland, Oregon, United States of America; Indiana University, United States of America

## Abstract

**Introduction:**

Acute kidney injury is a serious,sexually dimorphic perioperative complication, primarily attributed to hypoperfusion. We previously found that estradiol is renoprotective after cardiac arrest and cardiopulmonary resuscitation in ovariectomized female mice. Additionally, we found that neither estrogen receptor alpha nor beta mediated this effect. We hypothesized that the G protein estrogen receptor (GPR30) mediates the renoprotective effect of estrogen.

**Methods:**

Ovariectomized female and gonadally intact male wild-type and GPR30 gene-deleted mice were treated with either vehicle or 17β-estradiol for 7 days, then subjected to cardiac arrest and cardiopulmonary resuscitation. Twenty four hours later, serum creatinine and urea nitrogen were measured, and histologic renal injury was evaluated by unbiased stereology.

**Results:**

In both males and females, GPR30 gene deletion was associated with reduced serum creatinine regardless of treatment. Estrogen treatment of GPR30 gene-deleted males and females was associated with increased preprocedural weight. In ovariectomized female mice, estrogen treatment did not alter resuscitation, but was renoprotective regardless of GPR30 gene deletion. In males, estrogen reduced the time-to-resuscitate and epinephrine required. In wild-type male mice, serum creatinine was reduced, but neither serum urea nitrogen nor histologic outcomes were affected by estrogen treatment. In GPR30 gene-deleted males, estrogen did not alter renal outcomes. Similarly, renal injury was not affected by G1 therapy of ovariectomized female wild-type mice.

**Conclusion:**

Treatment with 17β-estradiol is renoprotective after whole-body ischemia-reperfusion in ovariectomized female mice irrespective of GPR30 gene deletion. Treatment with the GPR30 agonist G1 did not alter renal outcome in females. We conclude GPR30 does not mediate the renoprotective effect of estrogen in ovariectomized female mice. In males, estrogen therapy was not renoprotective. Estrogen treatment of GPR30 gene-deleted mice was associated with increased preprocedural weight in both sexes. Of significance to further investigation, GPR30 gene deletion was associated with reduced serum creatinine, regardless of treatment.

## Introduction

Acute Kidney Injury (AKI) is a common and serious complication of the perioperative period which is primarily attributed to whole-body hypoperfusion.[1] In the United States, approximately 150,000 patients per year experience perioperative AKI after noncardiac, nonvascular surgery – representing about 1% of all surgical procedures. In this group of patients, males are more likely to suffer AKI than females.[2] Interestingly, studies of cardiac and vascular patients have shown that males are *less* likely than females to develop AKI, and less likely to die or require dialysis.[3]–[8] Most women undergoing cardiac and vascular surgery are post or peri-menopausal because presentation of cardiac disease is delayed by the presence of estrogen. Declining ovarian function is associated with increased risk of AKI in cardiac surgery patients.[9] The observation that women have reduced risk of AKI in a population which includes premenopausal women (noncardiac, nonvascular surgery) but women who are mostly post- or peri-menopausal have increased risk of AKI is a suggestion that the presence of physiologic estrogen may be renoprotective in perioperative patients, and its absence may subject women to higher risk.[8] This observation has been borne out in animal studies, which have repeatedly demonstrated that males experience greater renal injury after ischemia than females.[10]–[12] Sex steroids have been implicated in this dimorphism with testosterone increasing injury and estrogen reducing it.[12]–[15]

Estrogen's actions are complex and governed through multiple mechanisms, which renders the potential use of estrogen as prevention or therapy for AKI problematic, and targeted therapy desirable. In general, estrogen's effects are believed to be mediated primarily through two cognate transcriptional receptors, α and β (ERα, ERβ).[16] A third, G protein-coupled estrogen receptor, GPR30, has recently generated significant interest and has been implicated in a number of estrogen's rapid effects.[17]–[20] Our recent work suggests that estrogen's renoprotective effect is not mediated by ERα or ERβ.[13] Accordingly, we hypothesized that GPR30 mediates estrogen's renoprotective action. To test this hypothesis, we employed an established whole-body ischemia model, cardiac arrest and resuscitation (CA/CPR). We subjected GPR30 gene-deleted mice (GPR30KO) to CA/CPR, measuring serum urea nitrogen (sUN), serum creatinine (sCr) and histologic outcomes 24 h after arrest. In order to tightly control estrogen levels in the normally cycling female mouse, we performed ovariectomy (OVX) on female mice prior to CA/CPR. Because estrogen may act differently in males and females, we assessed our intervention in both male and female animals.

## Methods

This study was conducted in accordance with the National Institutes of Health guidelines for the care and use of animals in research and all animal protocols were approved by the Oregon Health & Science University Institutional Animal Care and Use Committee. All animal experiments described were performed under general anesthesia.

### Animals and Experimental Groups

6–8 week old male and female C57BL/6 mice (Jackson Laboratory Sacramento, CA) served as wild-type (WT) controls. There were four groups each of male and ovariectomized (OVX) female mice, also 6–8 weeks old at the time of experimentation: (1) wild-type vehicle controls (WT-VEH, n = 8 female and 6 male), (2) wild-type 17β-estradiol treated (WT-EST, n = 13 female and 8 male), (3) GPR30-/- vehicle controls (GPR30KO-VEH, n = 7 female and 10 male), and (4) GPR30-/- 17β-estradiol-treated (GPR30KO-EST, n = 6 female and 10 male). There were 2 groups of female ovariectomized mice in the follow-on study of G1, vehicle-treated (VEH, n = 7), and G1-treated (G1,n = 9). All animals were fed the same diet, and housed in environmentally, and day-length controlled single-species housing.

### G Protein-Coupled Receptor 30 Knockout (GPR30KO) Mice

Breeding pairs were obtained from Dr. Halina Offner, Portland Veterans Affairs Medical Center. *Gper*, the gene encoding GPR30, was targeted in 129 SvEvTac embryonic stem (ES) cells by specific vector with Neo^r^ insertion as previously described.[21] This strain originated on a B6;129 background and has been backcrossed to C57BL/6J for at least 6 generations before breeding with homozygous breeders. Mice that are homozygous null for the targeted gene are viable and fertile and do not display any gross physical, immunological, reproductive, or neurological abnormalities. Foundation breeders for our colony were originally obtained through a material transfer agreement with Proctor & Gamble Pharmaceuticals (Mason, OH). Our colonies are currently maintained using a continuous homozygous trio breeding system so as to produce knockout mice as well as replacement breeders. These strains are genotyped by PCR as previously described.[21] A 3′-primer multiplex assay was developed and executed using Accuprime Supermix II amplification system and the following primers: for the GPR30 wild-type and knockout animals, the common *Gper* forward (5′-GAGCA CATCTGAGGA GCACT TTGCT GTCTC G-3′) primer is used, respectively, with the Neo reverse (5′-GGATC TCCTG TCATCTCACC TTGCT CCTGC C-3′) and wild-type *Gper* reverse primer (5′-GTGCC ACCAA CACCC AGCTC ACACA GC-3′). The common wild-type forward and wild-type reverse primers yield a 555 base pair band for the wild-type allele versus a 730 base pair band when the wild-type forward/Neo reverse primer is used for the targeted allele.

### Ovariectomy and Hormone Treatment

Ovariectomy and placement of a subcutaneous implant containing either vehicle, 17β-estradiol (6.3 µg total dose, prepared in our laboratory), or G1 (1.8 mg 21 day continuous release pellets, Innovative Research of America, Toledo OH) was performed under isoflurane anesthesia 7 days prior to CA/CPR. This dose of 17β-estradiol was chosen because we have previously shown that it reliably produces physiologic estradiol levels.[22] G1 is a high-affinity specific agonist of GPR30 with a *K_i_* of 11 nM and an EC_50_ of 2 nM [23] We chose our dose and method of administraton of G1 based on prior studies in our labs and others in which this dose was similarly effective to 17β-estradiol to produce protective effects.[24]–[26] Vehicle pellets were identical in all respect to drug-containing pellets excepting the treatment drug content, but the vehicle for 17β-estradiol and that for G1 were not identical. All surgeons performing ovariectomy in our laboratory have previously demonstrated proficiency measured by post-procedure serum estradiol levels.

### In-vivo whole-body ischemia-reperfusion

We performed normothermic CA/CPR as previously described.[13],[27] Mice were removed from cages in random order with respect to treatment by a surgeon who was blinded to hormonal status. After weighing, general anesthesia was induced with 4% isoflurane in 2∶1 air:oxygen mixture, and then maintained with 1-2% isoflurane. Animals were then placed in the supine position on a warming pad, under a warming lamp. A rectal temperature probe was inserted. The pad and lamp were controlled by a proportional-integral-derivative algorithm-controlled temperature controller (Digi-Sense, Cole Parmer, Vernon Hills IL), set to 37.0°C, and animal temperature maintained between 36.5 and 37.5°C. After tracheal intubation with a 22-ga Teflon catheter, animals were mechanically ventilated and subcutaneous electrocardiography (EKG) electrodes placed and secured. A PE-10 catheter was placed in the right jugular vein. After a 5 minute equilibration period, cardiac arrest (CA) was induced with 40 µL of 0.5 M potassium chloride intravenously. CA was confirmed by isoelectric EKG signal and absence of visible cardiac contraction on the chest wall. Isoflurane and mechanical ventilation were discontinued and the endotracheal tube disconnected. Nine and a half minutes after the onset of CA, the endotracheal tube was reconnected and mechanical ventilation resumed using 100% oxygen. Ten minutes after the onset of CA, chest compressions were initiated at a rate of 300/minute, measured by motion artifact on the EKG. Epinephrine, 5–16 µg in 0.9% sodium chloride solution (0.5–1.0 mL) was administered intravenously. Return of spontaneous circulation was confirmed by EKG and visible cardiac contractions on the chest wall. The jugular catheter was then removed and hemostasis obtained. Animals were extubated when their spontaneous respiratory rate was >60 breaths/minute, and placed in a recovery cage which was placed on a warming pad controlled at 37°C to prevent post-arrest hypothermia.

### Histologic Evaluation of Tubular Injury

Twenty-four hours after CA/CPR, deep general anesthesia was induced with isoflurane 4% in air:oxygen mixture. Upon cessation of spontaneous respiration, a clamshell thoracotomy was performed. After aspiration of the total accessible blood volume from the right ventricle, transcardial perfusion was performed via the apex of the left ventricle with 4% paraformaldehyde in 0.9% saline solution. Immediately thereafter, a postmortem midline laparotomy was carried out and the kidneys removed and preserved in 4% paraformaldehyde. Four 6 µM-thick sections were then made in the sagittal plane at equidistant locations starting at the renal pole. These were stained with Fluoro-Jade B (Histo-Chem, Jefferson, AK), which stains necrotic cells bright green. After slide preparation, an observer blinded to sex, strain and treatment assessed histologic damage according to the Cavalieri principal of unbiased stereology. We have previously described the specifics of unbiased stereology in this model.[13] Briefly, computer software (Visiopharm Integrator Software, Visiopharm, Hørsholm, Denmark) is interfaced with a 3 dimensional motion-controlled slide stage and digitizing microscope objective. The software randomly plots point grids over the microscopic image, which are the used to describe the reference space (i.e., the sagittal section) and the necrotic tubular cells which intersect plotted points within the reference space. The ratio of these two quantities is reported as a percentage (% tubular cell death, or Volume of Necrotic Tubules, VNC), which represents the fraction of all tubular cells which is necrotic.

### Evaluation of Serum Urea Nitrogen and Serum Creatinine

Blood drawn at the time of transcardial perfusion was placed in lithium heparin tubes. A point-of-care enzyme-coupled analyzer (Abaxis Medical Diagnostics, Union City, CA) was used to measure serum urea nitrogen (sUN) and serum creatinine (SCr). This device was chosen because it employs a creatinine amidohydrolase catalyzed assay, which is not subject to artifactual elevation of creatinine caused by chromogens in mouse serum.[28], [29]

### Statistical Analysis

Statistical significance was inferred from p<0.05. Correlation analysis was performed using Pearson's test with two-tailed p values. Two-group two-treatment analyses were performed using 2-way analysis of variance (ANOVA), followed by Holm-Sidak testing for analysis of individual difference in multiple group comparisons. Two-group comparisons were conducted using parametric, unpaired t-testing. Categorical outcome(survival) was assessed using χ-square. All results are reported as mean±standard error of the mean (SEM). Composite figures were created using ScientiFig.[30]

## Results

Pre-CA/CPR weight and resuscitation data are shown in table 1.

**Table 1:** Baseline and Resuscitation Data.

	*n*	CPR Time (s)	Epinephrine Dose (µg/mg body weight)	Pre-CA/CPR Body Weight (gm)	Mortality (%)
Female WT-VEH	8	95±11	0.40±0.03	21.7±0.4	77
Female WT-EST	13	94±8	0.40±0.02	22.1±0.2	61
Female GPR30KO-VEH	7	82±14	0.39±0.02	21.1±0.5	67
Female GPR30KO-EST	6	86±7	0.36±0.02	23.6±0.4	50
*p 17β-est treatment* *		0.8684	0.4363	0.0006	-
*p GPR30 deletion* *		0.3212	0.2228	.2038	-
*p interaction* *		0.8501	0.5125	.0160	-
*p (chi-square)*		-	-	-	0.27
Male WT-VEH	6	80±11	0.33±0.02	25.1±0.5	42
Male WT-EST	8	49±5	0.260.02	26.7±0.4	62
Male GPR30KO-VEH	9	75±9	0.33±0.02	25.7±0.4	47
Male GPR30KO-EST	10	51±4	0.25±0.01	27.8±0.5	23
*p17β-est treatment* *		0.0008	<0.0001	0.0009	-
*p GPR30 deletion* *		0.7966	0.9051	0.0662	-
*p interaction* *		0.6087	0.7811	0.5089	-
*p (chi-square)*		-	-	-	0.17
Female VEH	7	88±10	0.45±0.02	20.7±0.3	36
Female G1	9	100±7	0.49±0.03	21.1±0.3	47
*p (t-test)*		0.3	0.3	0.3	0.7

*p values shown for 2-way ANOVA. Post-ANOVA individual comparisons are within the text. Abbreviations: WT: Wild Type; GPR30KO: GPR30 gene-deleted mice; VEH: vehicle-treated mice; EST: 17β-estradiol-treated mice; sUN: serum urea nitrogen; sCr: serum creatinine; VNC: volume of necrotic tubules; CPR: cardiopulmonary resuscitation; CA/CPR:cardiac arrest and cardiopulmonary resuscitation.

### Estrogen Treatment Associates with Higher Post-Ovariectomy Body Weight in GPR30KO Mice

Estradiol treatment was associated with increased pre-CA/CPR body weight in GPR30KO female mice relative to vehicle-treated GPR30KO female mice (23.6±0.4 vs 21.1±0.5 gm, p = 0.0006), but estradiol treatment of female WT mice did not associate with increased weight (22.1±0.2 vs. 21.7±0.4 gm p = 0.3) While there was a nonsignificant trend toward increased weight in WT male mice treated with estradiol (1.6±0.7 gm, p = 0.06), estradiol treatment was associated with significantly greater pre-CA/CPR weight than vehicle treatment in male GPR30KO mice (GPR30KO-EST 27.8±0.5 gm, GPR30KO-VEH 25.7±0.4 gm, p = 0.004).

### Resuscitation Effects of Estrogen and GPR30 Gene Deletion Were Sex-Specific

There was no significant difference in mortality between paired experimental groups. Among female mice, neither GPR30 gene deletion nor estrogen treatment was associated with a significant difference in time-to-resuscitate (CPR time) or total dose of epinephrine required to achieve [31]return of spontaneous circulation. In male mice, however, CPR time and epinephrine dose were significantly reduced by estradiol treatment (p = 0.0008), there was no significant effect on resuscitation parameters from GPR30 gene deletion. Mean CPR time in males was reduced by estradiol treatment by 31.2 s in WT mice (p = 0.017)and by 23.7 s in GPR30KO (p = 0.018). The dose of epinephrine required to achieve return of spontaneous circulation was also reduced by estradiol treatment in male, but not female mice, by 0.07±0.02 µg/gm in WT (p = 0.01) and 0.08±0.02 µg/gm in GPR30KO mice (p = 0.002). This difference was still statistically significant following adjustment for the difference in weight found in estrogen-treated GPR30KO mice.

There were no significant differences in weight or resuscitation parameters between the G1- and vehicle-treated groups in the follow-on study.

Injection of potassium chloride caused immediate cardiac arrest in all mice. There was strong correlation between sUN,sCr and tubular histologic outcomes of CA/CPR. Both sUN and sCr correlated with the stereologic measure of volume of necrotic tubules (VNC) (n = 67, r = 0.69, p<0.0001 for sUN-VNC, r = 0.73, p<0.0001, for sCr-VNC).

### Estrogen Treatment Reduced Renal Injury in Females Irrespective of GPR30 Gene Deletion

**Figure 1:**
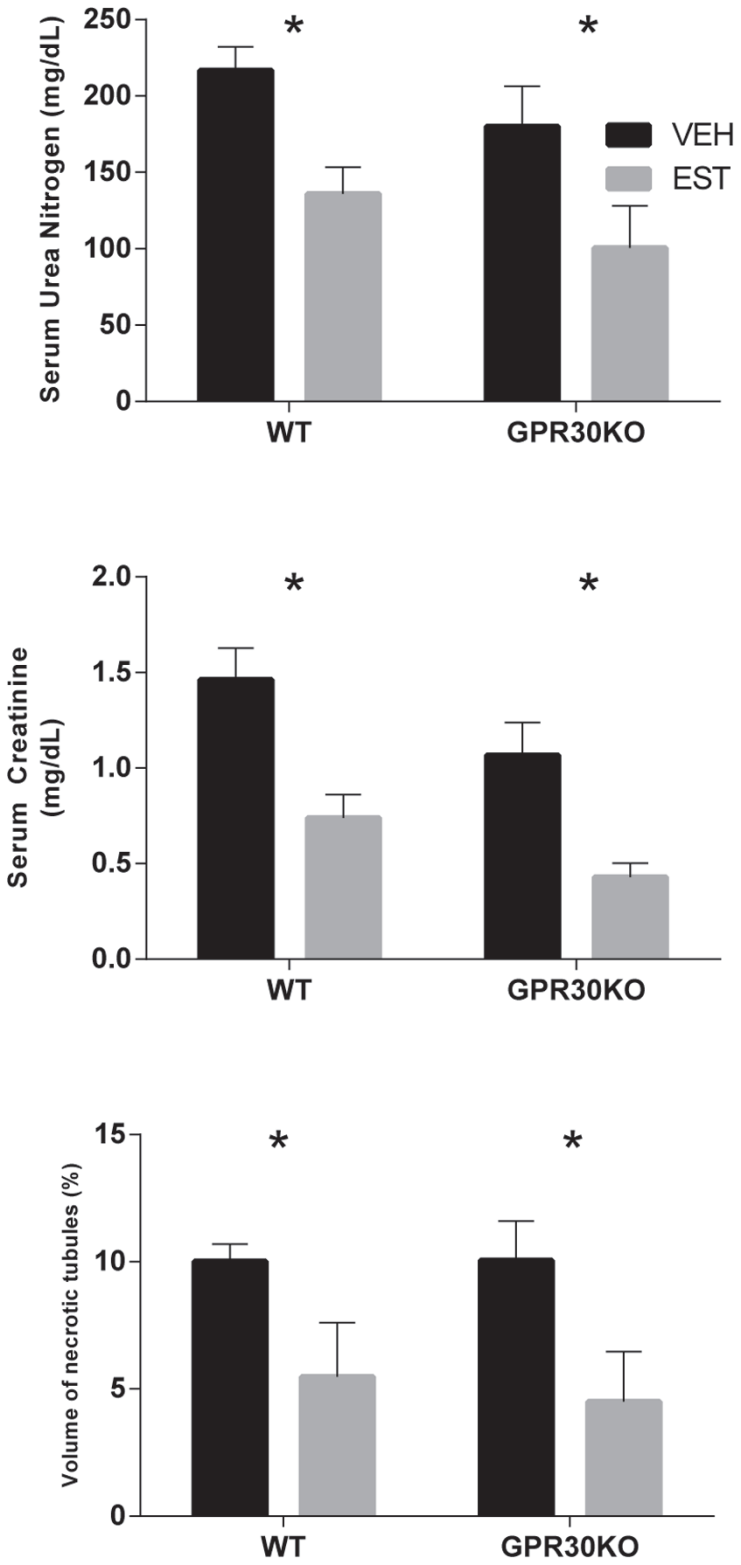
Serum urea nitrogen, serum creatinine, and histologic outcomes 24/CPR in ovariectomized female mice. Estradiol (EST) reduced renal injury in both wild-type (WT) and GPR30 gene-deleted (GPR30KO) mice, as evidenced by reduced serum urea nitrogen, serum creatinine, and volume fraction of necrotic tubules (% tubular cell death). (Mean±SEM, n = 8,13,7,6 respectively, for WT-VEH, WT-EST, GPR30KO-VEH, and GPR30KO-EST, *p<0.05).

In female mice, treatment with 17β-estradiol was renoprotective irrespective of the presence or absence of the GPR30 gene, and the interaction between GPR30KO and estradiol treatment was not significant. As shown in figure 1, sUN, sCr, and histopathologic injury 24 h after CA/CPR were significantly reduced by estradiol treatment in both WT and GPR30KO mice (WT-VEH vs. WT-EST: 217±15 vs. 135±18 mg/dL p = 0.013, 1.5±0.2 vs. 0.7±0.1 mg/dL p = 0.001, 10.0±0.7 vs. 5.5±0.6% p = 0.001, respectively for sUN, sCr, and VNC, GPR30KO VEH vs. EST: 180±26 vs. 100±28 mg/dL p = 0.027, 1.1±0.2 vs. 0.4±0.1 mg/dL p = 0.008, and 10.1±0.2 vs. 4.5±0.1% p = 0.001 respectively for sUN, sCr, and VNC). GPR30KO mice (both vehicle- and estradiol-treated) exhibited reduced sCr compared with WT mice (p = 0.021) but other indices of renal injury were not significantly altered overall by GPR30 gene deletion. This data is summarized in table 2, represented in figure 1.

**Table 2:** Summary of Quantitative Outcomes in Ovariectomized Female Mice.

	WT		GPR30KO	
	VEH	EST	VEH	EST
sUN (mg/dL)	217±15	135±18*	180±26	100±28*
sCr (mg/dL)	1.5±0.2	0.7±0.1*	1.1±0.2	0.4±0.1*
VNC (%)	10.0±0.7	5.5±0.6*	10.1±0.2	4.5±0.1*

*p<0.05, values shown for 2-way ANOVA, with respect to control (vehicle-treated) in same strain. Abbreviations: WT: Wild Type; GPR30KO: GPR30 gene-deleted mice; VEH: vehicle-treated mice; EST: 17β-estradiol-treated mice; sUN: serum urea nitrogen; sCr: serum creatinine; VNC: volume of necrotic tubules.

### GPR30 Gene Deletion Reduces Post-Ischemic Serum Creatinine in Males, but Estrogen Does Not Protect Males from Renal Injury Following CA/CPR

**Figure 2:**
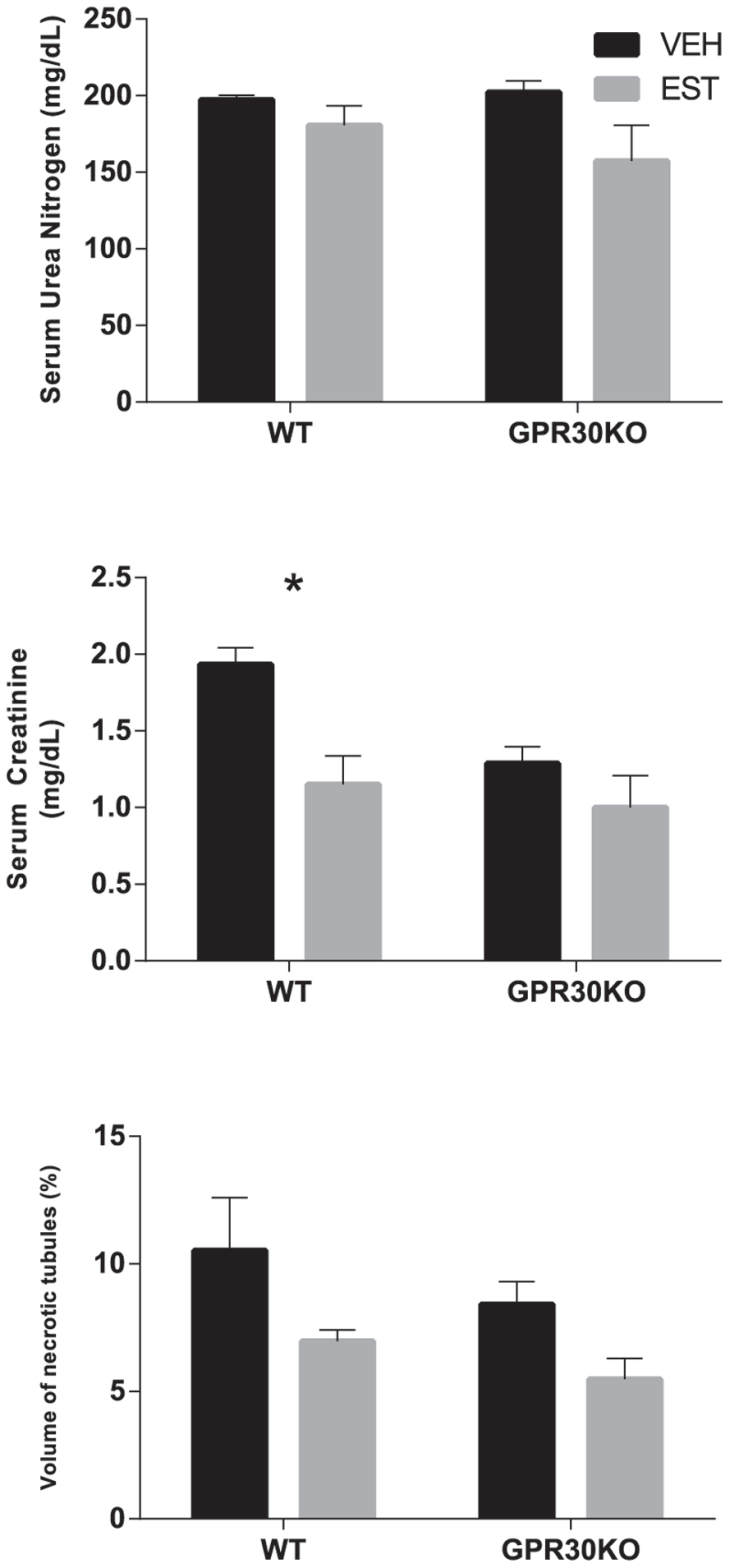
Serum urea nitrogen, serum creatinine, and histologic outcomes 24/CPR in male mice. Estradiol treatment significantly reduced serum creatinine, but not serum urea nitrogen, or tubular cell death in wild-type (WT) males. GPR30 gene deletion (GPR30KO) was associated with reduced serum creatine overall, but estrogen treatment did not reduce renal injury injury in GPR30 gene-deleted male mice. (Mean±SEM, n = 6,8,9,10 respectively, for WT-VEH, WT-EST, GPR30KO-VEH, and GPR30KO-EST, *p<0.05).

In male mice (as in females) GPR30 gene deletion caused a reduction in sCr in both vehicle- and estradiol treatment groups (p = 0.03 overall). Estradiol treatment significantly reduced sCR 24 h after CA/CPR in WT (1.9±0.1 mg/dL VEH vs. 1.2±0.2 mg/dL EST, p = 0.01) but not GPR30KO mice (1.3±0.1 mg/dL VEH vs. 1.0±0.2 mg/dL EST, p = 0.21). However, although there was a trend toward reduced injury in estradiol-treated mice there was no significant inter-group difference between vehicle- and estradiol- treated mice in sUN or histologic injury (WT VEH vs. EST 197±3 vs. 181±13 mg/dL, p = 0.5, 10.5±2.0 vs 7.0±0.4%, p = 0.7 respectively for sUN and VNC, GPR30KO VEH vs. EST 202±7 vs. 157±23 mg/dL, p = 0.08, 8.4±0.9 vs. 5.5±0.8%, p = 0.7 respectively for sUN and VNC). This data is summarized in table 3, represented in figure 2.

**Table 3:** Summary of Quantitative Outcomes in Male Mice.

	WT		GPR30KO	
	VEH	EST	VEH	EST
sUN (mg/dL)	197±3	181±13	202±7	157±23
sCr (mg/dL)	1.9±0.1	1.2±0.2*	1.3±0.1	1.0 ±0.2
VNC (%)	10.5±2.0	7.0±0.4	8.4±0.9	5.5±0.8

*p<0.05, values shown for 2-way ANOVA, with respect to control (vehicle-treated) in same strain. Abbreviations: WT: Wild Type; GPR30KO: GPR30 gene-deleted mice; VEH: vehicle-treated mice; EST: 17β-estradiol-treated mice; sUN: serum urea nitrogen; sCr: serum creatinine; VNC: volume of necrotic tubules.

### GPR30 Agonist G1 Does Not Mitigate Acute Kidney Injury following CA/CPR

**Figure 3:**
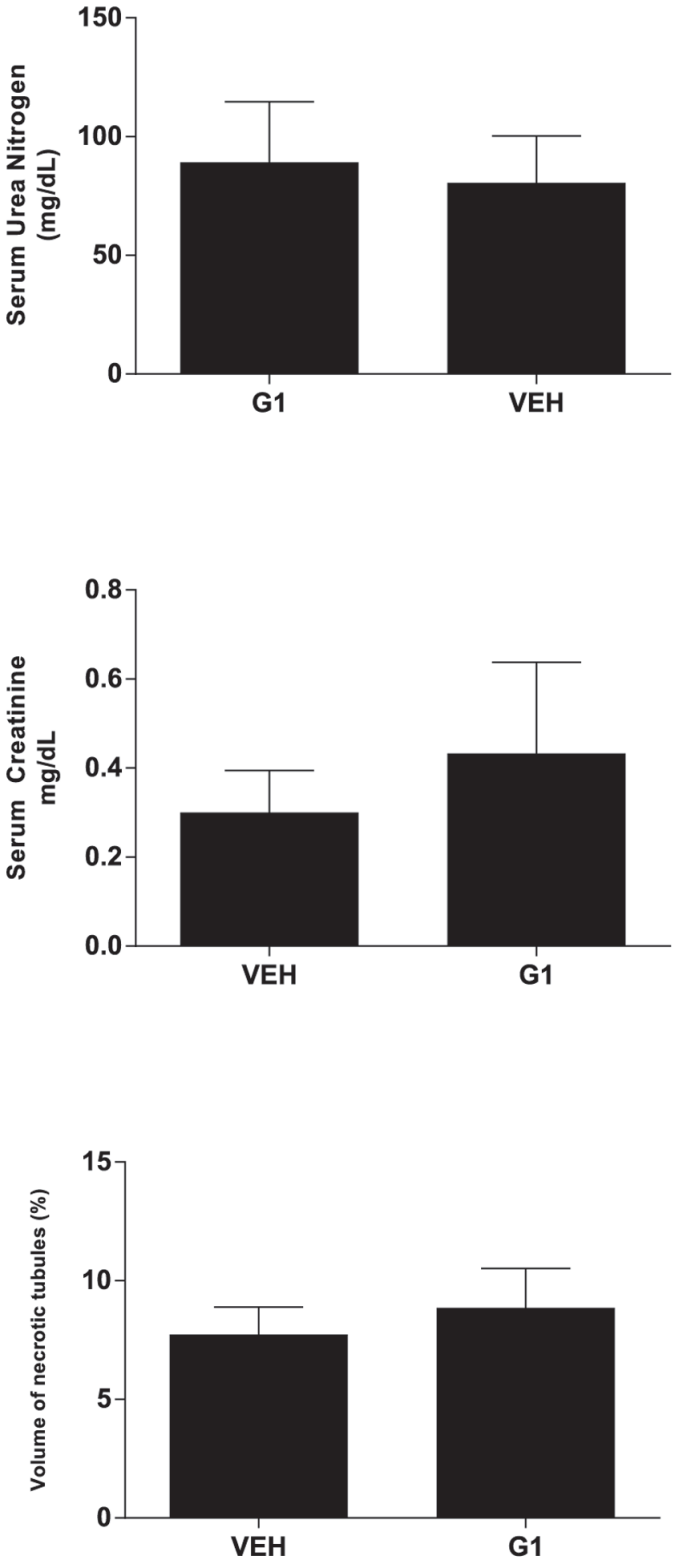
Serum urea nitrogen, serum creatinine, and histologic outcomes 24/CPR in ovariectomized female mice treated with either vehicle (VEH), or the GPR30-specific agonist G1. G1 treatment did not significantly alter renal injury after CA/CPR. (Mean±SEM, n = 7,9 respectively, for VEH and G1).

**Figure 4:**
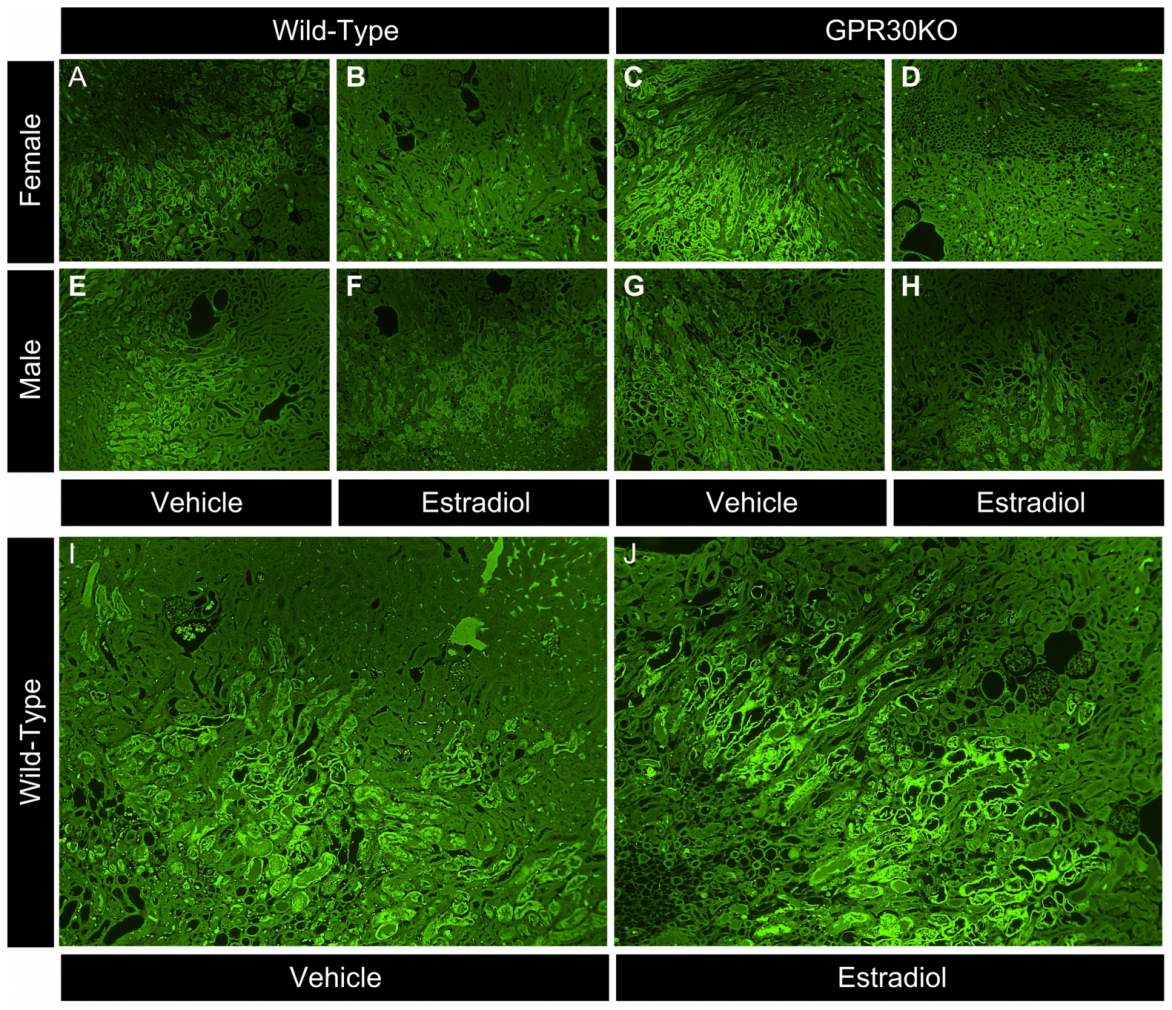
Representative Photomicrographs: Mouse kidneys were perfusion-fixed, removed, and stained with Flourojade-B, which stains early-necrotic cells bright green. Images taken of the cortico-medullary junction at 100× with no postprocessing other than pseudocolor simulating 532 nM flourescence. A)Female, wild-type vehicle control. B)Female, wild-type, estradiol-treated. C)Female, GPR30KO vehicle control. D)Female, GPR30KO, estradiol-treated. E)Male, wild-type vehicle control. F)Male, wild-type, estradiol-treated. G)Male, GPR30KO vehicle control. H)Male, GPR30KO, estradiol-treated. Second panel, wild-type vehicle vs. control. I)Wild-type, female vehicle control. J)Wild-type, female G1-treated.

Ovariectomized female mice administered the specific GPR30 agonist G1 suffered identical renal injury to vehicle controls (figure 3 with representative histology in figure 4,VEH vs. G1, sUN: 81±21 vs 89±26 mg/dL p = 0.8, sCr: 0.3±0.1 vs 0.4±0.2 mg/dL p = 0.6, VNC 7.7±1.2 vs. 9.0±1.7, p = 0.6).

## Discussion

The main finding of this study is that treatment with 17β-estradiol is renoprotective in ovariectomized female mice whether or not GPR30 is deleted. We also found that that 17β-estradiol treatment does not provide meaningful renoprotection in gonadally intact male mice despite salutory effects on resuscitation, and this is unaltered by GPR30 gene deletion. Activation of GPR30 with a pharmacologic agonist does not improve resuscitation parameters or ameliorate renal injury 24 hours after whole-body ischemia-reperfusion in ovariectomized females. GPR30 gene deletion was associated with significant metabolic differences: a reduced overall creatinine in both males and females, and an increased overall body mass in treated relative to untreated animals after exposure to 17β-estradiol in both sexes.

### Treatment with 17β-estradiol is renoprotective in ovariectomized female mice whether or not GPR30 is deleted and activation of GPR30 with a pharmacologic agonist does not improve resuscitation parameters or ameliorate renal injury

In previous experiments using the same methodology we found that treatment with 17β-estradiol was protective in both α-estrogen receptor gene-deleted (αERKO) and β-estrogen receptor gene-deleted mice.[13] GPR30 is involved in protection from ischemia-reperfusion injury in neurons[26], [32] and in cardiomyocytes.[33],[34] Work in chronic kidney disease models has suggested that GPR30 agonists exert protective effects.[35] Distal convoluted tubule[36] and glomerular endothelial cells express GPR30, and *in vitro* estrogen amelioration of ischemia-reperfusion injury is ablated by antagonism of GPR30 in glomerular endothelial cells.[37] We thus began the current study with the hypothesis that GPR30, a third estrogen receptor, acting either in glomerular endothelial cells, or directly within the proximal tubular epithelium, was the mediating receptor for the protective effect. Our results, in genetically-altered mice and mice treated with a GPR30 pharmacologic agonist suggest that GPR30 does not mediate the robust renoprotective effect of estrogen in ovariectomized female mice subjected to CA/CPR. The use of paired genetic and pharmacologic manipulation makes the conclusion that this is artifactual to experimental design unlikely. Our conclusion is limited to a single dose of estrogen, designed to replicate physiologic levels, and to a single model of ischemia-reperfusion injury. The data are robust however, and suggest one of two possibilities – either an an additional receptor or nonreceptor effect is involved, or the mechanism may be triggered by multiple receptors. In addition to receptor-mediated effects, estrogen effects, particularly rapid effects in ischemia, have been postulated to be mediated by nonreceptor effects.[38]–[40] Putative mechanisms of nonreceptor effects include antioxidant and lipid peroxidation inhibition effects based on structure-activity assays of estrogenic compounds with similar protective activity[41], [42]. Two putative fourth cognate receptor candidates have been described: Gq-mER,[43] and ER-X.[44], [45] Gq-mER, in particular mediates at least one rapid signaling pathway which estrogen triggers in ERα/β gene-deleted and GPR30 gene-deleted mice.[46] We did not assess these potential pathways. A mechanism mediated by multiple receptors is a second potential implication of our study. Multiple receptors converging to a single endpoint would suggest a highly-conserved, organism preserving strategy. This is consistent with the need to survive ischemia-reperfusion. Estrogen signals lipogenesis, a long-term survival mechanism through both ERα and ERβ, suggesting that it is possible that other important mechanisms associated with survival could be modulated by more than one receptor simultaneously.[47] The current study did not address this possibility, but future study using a combination of nonspecific pharmacologic inhibition and individual receptor gene-deletion could help elucidate this complex hypothesis.

### 17β-estradiol treatment does not provide meaningful renoprotection in gonadally intact male mice despite salutory effects on resuscitation

Although estrogen treatment was associated with reduced creatinine in WT males, serum urea nitrogen and histologic outcome were not affected. We conclude that estrogen treatment did not provide meaningful renal protection in WT males. In our previous study, we noted that estrogen did not significantly alter resuscitative time or epinephrine requirement, post-arrest blood pressure, or post-arrest renal blood flow in ovariectomized female mice.[13], [37] The present study confirms the our prior finding of renoprotection unrelated to time-to-resuscitate or epinephrine dose in females, but adds a reciprocal finding that 17β-estradiol treatment does not provide postischemic renoprotection in intact male mice, despite significantly improving time-to-resuscitate and epinephrine requirement in both WT and GPR30KO mice. Because we did not find a renoprotective effect of estrogen in male mice, we did not pursue further study with the GPR30 pharmacologic agonist, G1, in males. Sexual dimorphism in renal ischemic response to estrogen has been previously reported. Park et al found estrogen renoprotection in gonadally intact males, but little effect in ovariectomized females in a renal pedicle clamp model which produced minimal injury in females.[12] Muller et al found reduced survival in male rats subjected renal ischemia-reperfusion injury which was reversed with estradiol treatment, but was underpowered to detect renoprotection by estrogen in males. [11] More recently a sympatholytic effect of estrogen has been implicated in the worsened AKI of males. Tanaka et al found that male plasma norepinephrine was more than double that of females and male renal injury was worse after renal ischemia-reperfusion. Interference with the outflow of norepinephrine reduced sUN, sCr, and histologic renal injury in both males and females, and ovariectomy ablated both the difference in symathetic response and renal injury.[48] This estrogen sympatholysis may provide a partial explanation for the resuscitation metric sexual dimorphism seen in the present study. We did not find significant renoprotection by estrogen administration to males, however. We speculate our observation that estrogen improves resuscitation time-to-resuscitate and epinephrine dose, but not renal outcomes in males, but improves renal outcomes but not time-to-resuscitate and epinephrine dosein females may signal the vascular effects of estrogen are not critical to the renoprotective effect. This is in accordance with our prior work, in which we found no estrogen-mediated effect on postresuscitation regional renal cortical blood flow,[37] and supports a role for estrogen in postischemic signaling, in accordance with work suggesting estrogen suppresses renal endothelin-1 production,[11], [49] activates the PI3K/Akt pathway and phosphorylates eNOS.,[50] This conclusion is limited by several factors. First, we studied a single dose of estrogen designed to produce normal-female physiologic levels. The relevance of this dose to male animals is unclear, and it is possible that a different dose would have produced significant protection. In neurologic outcomes of the same ischemia-reperfusion model, estrogen protection is dose-dependent, with doses outside the optimal range causing harm.[51] Second, we employed CA/CPR, a whole-body ischemia-reperfusion model, while the referenced studies were performed in focal-renal ischemia reperfusion models which employ clamp-occlusion of the entire renal pedicle. These models have recently shown to induce distinct outcomes and generalizing from pedicle occlusion to whole body ischemia-reperfusion could be erroneous.[52]

### GPR30 gene deletion was associated with metabolic effects, with reduced overall creatinine in both males and females, and increased overall body mass in response to 17β-estradiol treatment in both sexes

Estrogen is a pleiotropic molecule with wide-ranging metabolic effects. Several groups have investigated the effects of GPR30 gene deletion on body mass, bone mass, and lipid handling. In general, GPR30 gene-deleted male and female animals have increased body weight compared with WT mice.[53]–[55] Using micro-computed tomography, Ford et al found this was due to increased bone mass as well as adiposity[55] while Sharma et al attribute the difference largely to adiposity, having found no difference in tibia-length measurements.[54] To our knowledge, no prior study has reported on body weight following estrogen administration in ovariectomized female or gonaldally-intact male GPR30KO mice. We measure body weight immediately prior to CA/CPR and our finding was unexpected. We observed a small increment in body mass in estrogen-treated GPR30KO mice relative to vehicle-treated animals, (11% of overall body mass in females, 8% in males), but this is statistically significant. As this increment is small and occurred over 7d in young mice, it may be due to bone mass effects, as increased growth in other compartments (adiposity, fluid retention) would be expected to be larger in magnitude over this time period. One possible explanation is that estrogen administration reduces body mass by an unknown GPR30-dependent mechanism which is unmasked by GPR30 gene deletion. Another, related possibility involves the well-known ovariectomy-associated increase in weight in females. Since all female mice in our study were ovariectomized, and ovariectomy is associated with increased weight, it is possible that estrogen treatment reduced the body weight of WT ovariectomized females with intact GPR30, but not that of GPR30KO mice. This alone would not explain the difference observed in male mice. The hypothesis under which we designed experiments was not related to body weight, and we did not therefore measure body weight prior to ovariectomy. In retrospect, this data would be helpful to interpret the meaning of this novel observation, which we believe warrants further investigation. Finally, the reduction in serum creatinine associated with GPR30 gene deletion suggests either increased creatinine clearance or reduced muscle mass. The former is supported by the observation of Lindsey and associates who observed increased creatinine clearance in hypertensive rats treated with G1.[56] We did not measure creatinine clearance in our study, nor did we measure body mass prior to estrogen treatment. Again, we believe this observation, which our study was not designed to detect, warrants further investigation.

## Conclusions

We conclude that estrogen-mediated renoprotection is robust to the deletion of GPR30, does not depend on acute improvements in time-to-resuscitate and epinephrine doseresultsand does not occur with specific agonist activation of the GPR30 receptor in ovariectomized female mice. In males, although estrogen treatment is associated with improved time-to-resuscitate and epinephrine dose, treatment with physiologic doses of 17β-estradiol was not renoprotective. Strengths of our study include a clinically-relevant whole-body ischemia reperfusion model and use of both genetic and pharmacologic manipulation of the same receptor. Our study is limited in that we evaluated renal protection in a single ischemia-reperfusion model, and our conclusions are limited to those that can be drawn from a mouse model. We are currently pursuing further study of this important phenomenon.

## Supporting Information

Checklist S1 Arrive checklist.
(PDF)
